# Heliotherapy for Neonatal Hyperbilirubinemia in Southwest, Nigeria: A Baseline Pre-Intervention Study

**DOI:** 10.1371/journal.pone.0151375

**Published:** 2016-03-22

**Authors:** Abieyuwa A. Emokpae, Cecilia A. Mabogunje, Zainab O. Imam, Bolajoko O. Olusanya

**Affiliations:** 1 Massey Street Children’s Hospital, Lagos, Nigeria; 2 Centre for Healthy Start Initiative, 286A Corporation Drive, Dolphin Estate, Ikoyi, Lagos, Nigeria; Hôpital Robert Debré, FRANCE

## Abstract

**Background:**

A novel filtered-sunlight phototherapy (FSPT) device has been demonstrated to be safe and efficacious for treating infants with neonatal jaundice in resource-constrained tropical settings. We set out to provide baseline data for evaluating the clinical impact of this device in a referral pediatric hospital.

**Methods:**

We reviewed the medical records of infants admitted for neonatal hyperbilirubinemia in an inner-city Children’s Hospital in Lagos, between January 2012 and December 2014 to determine the pattern, treatment and outcomes during the pre-intervention period. Factors associated with adverse outcomes were identified through multivariable logistic regression.

**Results:**

Of the 5,229 neonatal admissions over the period, a total of 1,153 (22.1%) were admitted for neonatal hyperbilirubinemia. Complete records for 1,118 infants were available for analysis. The incidence of acute bilirubin encephalopathy (ABE) and exchange transfusion (ET) were 17.0% (95% CI: 14.9%–19.3%) and 31.5% (95% CI: 28.8%–34.3%) respectively. A total of 61 (5.5%, 95% CI: 4.3%–6.9%) of the jaundiced infants died. Weight on admission, peak total serum bilirubin (TSB), sepsis and exposure to hemolytic products were predictive of ABE, while age on admission, peak TSB, ABO incompatibility and ABE were predictive of ET. Rhesus incompatibility, asphyxia, exposure to hemolytic substances and ABE were associated with elevated mortality risk, while ET was a protective factor. Lack of routine irradiance monitoring and steady energy supply were frequent challenges for conventional blue-light phototherapy.

**Conclusions:**

Severe hyperbilirubinemia is associated with high rates of ABE and ET in this setting, and remains a significant contributor to neonatal admissions and mortality. To be impactful, FSPT, complemented with improved diagnostic facilities, should effectively curtail jaundice-related adverse outcomes in this and comparable settings.

## Introduction

Some degree of neonatal jaundice is a benign, transitional phenomenon that affects 60%–80% of newborns worldwide [1,2]. In a proportion of infants, jaundice may become severe, typically with total plasma/serum bilirubin (TSB) ≥20mg/dL or 342μmol/L, necessitating prompt hospitalization for phototherapy and/or exchange transfusion (ET) to halt potential progression to acute bilirubin encephalopathy (ABE) or kernicterus [2–4]. Timely detection of infants at risk of severe hyperbilirubinemia and the optimization of conventional blue-light phototherapy (CPT) in particular, has significantly curtailed the need for ET in developed nations [5–7]. However, in many low- and middle-income countries (LMICs), late presentation and lack of effective or intensive phototherapy frequently accounts for excessive rates of avoidable ET, ABE and kernicterus with substantial risks of mortality and long-term morbidity [8–11]. Special/intensive care baby units in many LMICs either lack phototherapy devices due to financial constraints [12–14], or have ineffective phototherapy as a result of erratic power supply, inadequate skin exposure from overcrowding with multiple infants placed under a single device, sub-optimal irradiance levels, and poor device maintenance [15–17].

As part of the efforts to address these constraints, a low-cost, low-maintenance, filtered-sunlight phototherapy (FSPT) canopy that requires no electricity has been successfully piloted for tropical LMICs [18]. The safety and efficacy of the device has been shown to be comparable to CPT in treating infants with predominantly mild-to-moderate hyperbilirubinemia (TSB ≤15 mg/dL or 256 μmol/L) prior to hospital discharge [19]. Mothers have also expressed satisfaction with the device as an alternative where CPT is not available [20]. However, the overall impact of FSPT in reducing ET rates and hyperbilirubinemia-related mortality in a referral hospital has not been reported.

**Fig 1:**
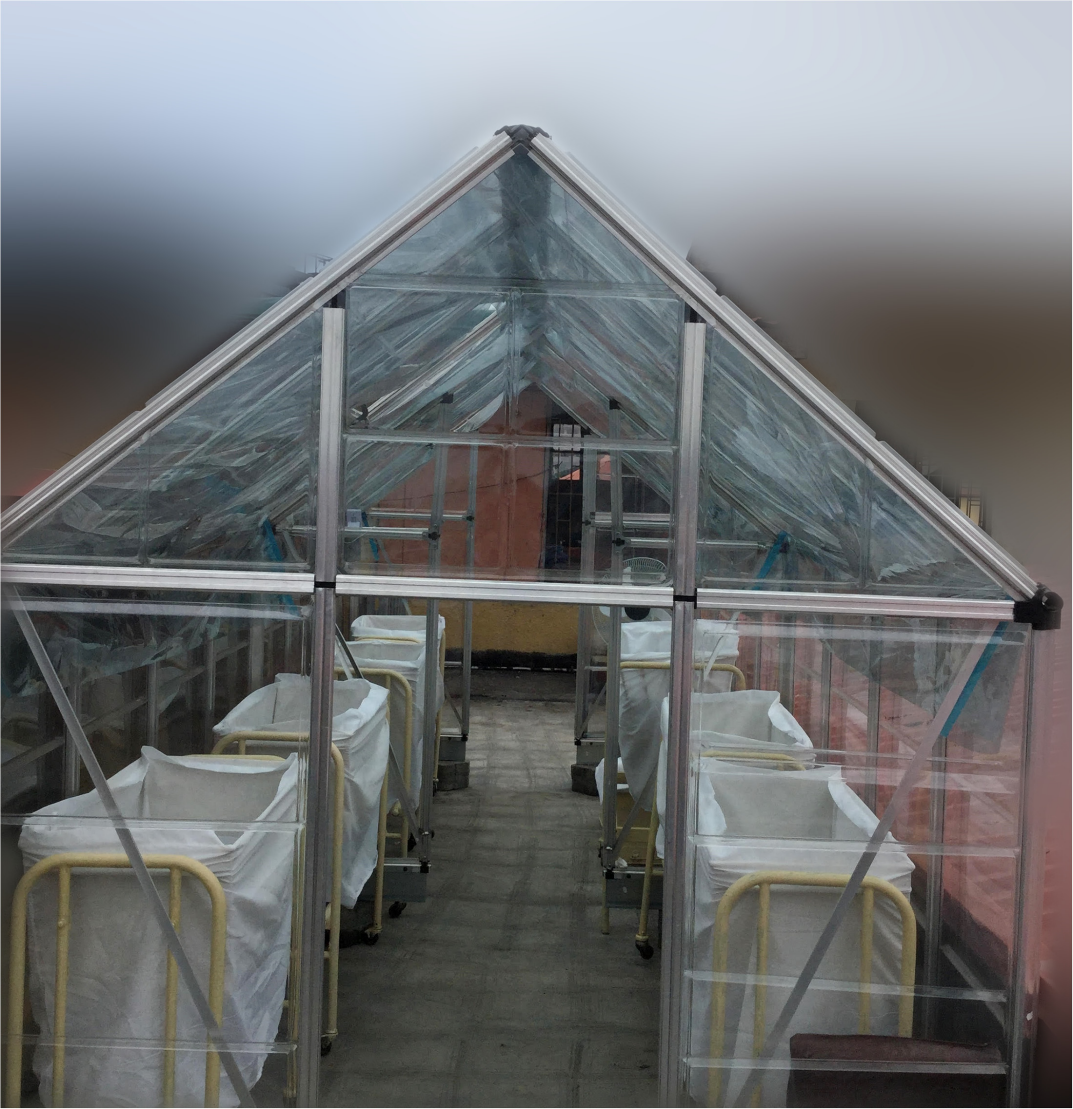
Proposed filtered sunlight phototherapy enclosure with embedded films.

We set out to report the baseline status and outcomes of infants who were treated for hyperbilirubinemia in the pre-intervention period in our pediatric referral hospital as basis for assessing the potential impact of *an* FSPT enclosure (Fig 1). The secondary aim was to identify the risk factors for these outcomes and other service-related issues that typically need to be addressed to optimize the benefits of the proposed intervention.

## Methods

This observational chart review was conducted at Massey Street Children’s Hospital (MSCH) in inner-city Lagos, Southwest, Nigeria. MSCH is a 100-bed, state-owned referral hospital that provides critical pediatric care to several private and public hospitals within and outside its catchment area. It is the first children’s specialist hospital in Nigeria. We manually reviewed the medical records of all neonates who were admitted for hyperbilirubinemia or developed hyperbilirubinemia while on admission from January 2012 to December 2014.

The standard protocol in the hospital was to place all infants presenting with severe jaundice (evidenced by generalized icterus extending to the palms and soles, or features of ABE) under phototherapy while the TSB was measured within one to three hours using the Advanced Bilirubin Stat-Analyzer (Model BR 2) (Advanced Instruments Inc, Norwood, CA). Blood samples were obtained from child for immediate laboratory investigations to determine baseline complete blood count, total and indirect serum bilirubin based on standard procedures [21]. Blood grouping and cross-matching were also determined from mother and child blood samples. When indicated, clinical diagnosis of infection (proven by positive blood culture and/or signs and symptoms consistent with sepsis) and neonatal encephalopathy (frequently termed as “birth asphyxia”) was routinely documented based on World Health Organization (WHO) guidelines for management of illnesses in resource-limited settings [22]. Since all infants were out-born, diagnosis of neonatal encephalopathy was based on birth history and physical state of the infant on admission. Tests for direct antiglobulin and glucose 6-phosphate-dehydrogenase (G6PD) status were not available during the study period. However, use of common hemolytic substances such as menthol-based baby products and naphthalene (camphor) balls was routinely elicited and documented on admission [23,24].

Because of the high prevalence of G6PD deficiency in this population [23], phototherapy was commenced at approximately TSB ≥12 mg/dL (204 μmol/L) in otherwise healthy normal weight (≥2500 g) babies [24]. The exchange transfusion level, as in most other local centers, was typically set at TSB ≥20 mg/dL (340 μmol/L) in apparently healthy near-term and term infants (gestational age ≥35 weeks or birthweight >2.2 kg) and at TSB <20 mg/dL in very ill term infants with or without features of ABE [24]. ET was indicated in preterm (gestational age <35 weeks) or low birthweight (≤2.2 kg) infants at TSB >10 mg/dL per kilogram body weight. The procedure was initiated immediately following the clinical diagnosis of ABE or kernicterus; or whenever phototherapy did not significantly lower TSB levels below the exchange threshold for postnatal age. ABE was indicated by any of the following symptoms in severely jaundiced infants: poor sucking, decreased alertness/lethargy, high-pitched cry, hypertonia of the extensor muscles, hypotonia, apnea, fever, retrocollis, opisthotonus or seizures [25]. The term “kernicterus” was reserved for chronic and permanent clinical sequelae of bilirubin toxicity, typically diagnosed after the first weeks of life. The hospital protocol mandated double-volume ET using fresh whole blood with the push-pull technique. Prophylactic antibiotics were routinely administered for all infants undergoing ET because of the risk of infection from the procedure.

We extracted the following data for each patient: gender, age and weight on admission or diagnosis of hyperbilirubinemia, peak total serum bilirubin (TSB), diagnosis of acute bilirubin encephalopathy (ABE), treatment with ET and outcome. Results of laboratory investigations were reviewed for positive history of ABO incompatibility (mother with O blood group and newborn with A, B or AB blood group) and Rhesus isoimmunization (Rhesus negative mother and Rhesus positive newborn). Other relevant medical conditions were also extracted.

### Ethics statement

This study was conducted under the institutional ethical approval (Reference No: SHMB/728/Vol. VI, dated 23 January 2015) from Lagos State Health Service Commission, the Ethics Review Board of all hospitals owned and managed by the Lagos State Government of Nigeria. As a retrospective chart review without prior individual consent, all patient records were anonymized and de-indentified prior to analysis.

### Statistical analysis

Cross-tabulation of the variables for each year was done to provide a descriptive overview of the study participants. All infants with incomplete data were excluded. Continuous variables were presented as mean and standard deviation (SD) for normally distributed data, or median (25th–75th percentiles) for skewed data, as assessed by the Kolmogorov-Smirnov, and Shapiro Wilks tests. Univariate associations between the variables and ABE, ET and mortality were explored with Pearson’s chi-square or Fisher’s exact tests (categorical variables) and with Student’s *t*-test (continuous variables). Strength of association was estimated by odds ratios (OR) and the corresponding 95% confidence intervals (CI) as an approximation of the relative risk. Statistical significance was set at a critical level of p<0.05. Factors significantly associated with each outcome in the univariate analysis were entered into the respective multivariable logistic regression model. Factors that did not significantly (p≥0.05) contribute to the model were eliminated by backward stepwise method to derive adjusted odds ratios (AOR). The model for ET was adjusted for ABE while that of mortality was adjusted for both ABE and ET. Model performance was assessed by the c-statistic (as shown by the area under the receiver operating characteristic curve) [26]. There were no a priori hypotheses for interaction terms so these were not investigated. We also examined the relationship between infants with at least one risk factor for neurotoxicity (ABO incompatibility, Rhesus incompatibility, sepsis and/or perinatal asphyxia) and ABE, ET and neonatal mortality. IBM SPSS Statistics for Windows software, Version 21.0 (Armonk, NY: IBM Corporation, USA) was used for all statistical analyses.

## Results

The total neonatal admissions for the three year period was 5229 out of which 1153 (22.1%) were admitted for hyperbilirubinemia. Thirty five infants were excluded because of incomplete data, leaving a total of 1118 infants for the present analysis (Table 1). The proportion of infants admitted for hyperbilirubinemia increased from 21.5% in 2012 to 24.9% in 2013, but declined to 17.4% in 2014. Male infants with hyperbilirubinemia were consistently almost twice as many as their female counterparts. The age on admission ranged from 1 day to 17 days with an overall median of 5 (IQR: 3–5) days. On average, over three-quarters of the infants presented in the first week of life, and about two-thirds between days 3 and 7. Infants with birth weight of 2.2kg or less averaged 8.7% of all infants admitted for hyperbilirubinemia. Approximately 30% of infants were born outside health facilities, mostly in residential homes and traditional maternity homes (data not shown). The peak TSB ranged from 2.1 mg/dL to 67 mg/dL with an overall median of 16.1 (IQR:12.0–22.6) mg/dL. The incidence of ABE and ET averaged 17% (95% CI: 14.9%–19.3%) and 31.5% (95% CI: 28.8%–34.3%) of infants admitted for hyperbilirubinemia respectively. Some 13.9% (49/352) of the infants had repeat ET. An average of 118 exchange transfusions were done per year, translating to some 2 to 3 ETs per week. Almost half (46.0%) of infants who received ET had no ABE. About half (51.1%) of all infants had at least one risk factor for neurotoxicity. The average mortality rate was 5.5% (95% CI: 4.3%–6.9%). There were no significant differences in these factors year-on-year, except for the significant drop in the number of parent-reported use of hemolytic substances as from 2012 (p<0.0001) and the sharp rise in mortality rate from 4.3% in 2013 to 8.2% in 2014 (p = 0.028). Cephalhematoma was reported only in four infants, biliary atresia in one infant, and intracranial hemorrhage in one infant during the study period.

**Table 1:** Profile of infants admitted for significant neonatal hyperbilirubinemia.

Category	2012	2013	2014	Total
	n = 348 (%)	n = 464 (%)	n = 306 (%)	n = 1118
**Gender**				
** Male**	234 (67.2)	289 (62.3)	194 (63.4)	717 (64.1)
** Female**	114 (32.8)	175 (37.7)	112 (36.6)	401 (35.9)
**Age on admission (days)**				
** 1–2**	40 (11.5)	98 (21.1)	54 (17.6)	192 (17.2)
** 3–7**	252 (72.4)	293 (63.1)	203 (66.3)	748 (66.9)
** 8–14**	56 (16.1)	73 (15.7)	48 (15.7)	177 (15.8)
** Above 14**	0 (0)	0 (0)	1 (0.3)	1 (0.1)
** Median (Interquartile range)**	5.0 (3.0–7.0)	4.0 (3.0–6.0)	4.0 (3.0–6.0)	5.0 (3.0–6.0)
**Weight on admission (kg)**				
** 2.2 kg or less**	30 (8.6)	36 (7.8)	31 (10.1)	97 (8.7)
** Above 2.2kg**	318 (91.4)	428 (92.2)	275 (89.9)	1021 (91.3)
** Median (Interquartile range)**	2.8 (2.5–3.3)	2.9 (2.5–3.3)	2.9 (2.6–3.2)	2.9 (2.5–3.3)
**Peak total bilirubin (mg/dL)**				
** <16**	162 (46.6)	232 (49.8)	134 (43.8)	527 (47.1)
** 16–20**	84 (24.1)	97 (20.9)	66 (21.6)	247 (22.1)
** 21–25**	44 (12.6)	53 (11.4)	43 (14.1)	140 (12.5)
** 26–30**	30 (8.6)	31 (6.7)	22 (7.2)	83 (7.4)
** >30**	28 (8.0)	52 (11.2)	41 (13.4)	121 (10.8)
** Median (Interquartile range)**	16.2 (11.3–21.9)	15.5 (11.6–21.9)	16.7 (12.6–23.7)	16.1 (12.0–22.6)
**Parent reported exposure to hemolytic agents/chemicals**				
** No**	329 (94.5)	461 (99.4)	298 (97.4)	1088 (97.3)
** Yes**	19 (5.5)	3 (0.6)	8 (2.6)	30 (2.7)
**Acute bilirubin encephalopathy**				
** No**	284 (81.6)	393 (84.7)	251 (82.0)	928 (83.0)
** Yes**	64 (18.4)	71 (15.3)	55 (18.0)	190 (17.0)
**Had exchange transfusion**				
** No**	247 (71.0)	321 (69.2)	198 (64.7)	766 (68.5)
** Yes**	101 (29.0)	143 (30.8)	108 (35.3)	352 (31.5)
**Had repeat exchange transfusion**				
** No**	334 (96.0)	439 (94.6)	296 (96.7)	1069 (95.6)
** Yes**	14 (4.0)	25 (30.8)	10 (3.3)	49 (4.4)
**Mortality**				
** No**	332 (95.4)	444 (95.7)	281 (91.8)	1057 (94.5)
** Yes**	16 (4.6)	20 (4.3)	25 (8.2)	61 (5.5)
**Neonatal admissions**	1612	1863	1754	5229

Factors associated with ABE are presented in Table 2. Of all infants with ABE 30 (15.8%) had peak TSB ≤20 mg/dL. All factors were significantly associated with ABE in the univariate analysis. However, after adjusting for the confounding effects of covariates, weight on admission (p = 0.041), peak TSB level (p<0.0001), sepsis (p<0.0001) and exposure to hemolytic substances (p = 0.012) were predicitive of ABE. Risk of ABE was elevated with increasing peak TSB, and highest at TSB >30 mg/dL (AOR: 306.13, 95%CI:105.47–888.59). The regression model had a strong discriminatory power as shown by the c-statistic estimate of 0.902 (95% CI: 0.879–0.925), suggesting that these factors can accurately predict ABE 90% of the time in this population.

**Table 2:** Factors associated with acute bilirubin encephalopathy.

Factors	No ABE	ABE		
	n = 928 (%)	n = 190 (%)	Unadjusted OR (95% CI)	Adjusted OR (95% CI)
**Gender**				
** Male**	580 (62.5)	137 (72.1)	1.55 (1.10–2.19)	
** Female**	348 (37.5)	53 (27.9)	Reference	
**Age on admission (days)**				
** 1–2**	183 (19.7)	9 (4.7)	Reference	
** 3–7**	601 (64.8)	147 (77.4)	4.97 (2.49–9.95)	
** 8–21**	144 (15.5)	34 (17.9)	4.80 (2.23–10.33)	
** Median (Interquartile range)**	4.0 (3.0–6.0)	5.0 (4.0–6.0)		
**Weight on admission (kg)**				p = 0.041
** 2.2 kg or less**	73 (7.9)	24 (12.6)	1.69 (1.04–2.77)	1.92 (1.03–3.60)
** Above 2.2kg**	855 (92.1)	166 (87.4)	Reference	Reference
** Median (Interquartile range)**	2.9 (2.5–3.3)	2.8 (2.5–3.1)		
**Peak total bilirubin (mg/dL)**				p<0.0001
** <16**	523 (56.4)	4 (2.1)	Reference	Reference
** 16–20**	221 (23.8)	26 (13.7)	15.38 (5.31–44.59)	15.82 (5.43–46.04)
** 21–30**	148 (15.9)	75 (39.5)	66.26 (23.84–184.15)	62.33 (22.27–174.40)
** >30**	36 (3.9)	85 (44.7)	308.72 (107.16–889.39)	306.13 (105.47–888.59)
** Median (Interquartile range)**	14.6 (11.2–18.9)	29.6 (23.1–34.8)		
**ABO incompatibility**				
** No**	806 (86.9)	144 (75.8)	Reference	
** Yes**	122 (13.1)	46 (24.2)	2.11 (1.44–3.09)	
**Rhesus incompatibility**				
** No**	901 (97.1)	176 (92.5)	Reference	
** Yes**	27 (2.9)	14 (7.4)	2.65 (1.37–5.16)	
**Sepsis**				p<0.0001
** No**	655 (70.6)	105 (55.3)	Reference	Reference
** Yes**	273 (29.4)	85 (44.7)	1.94 (1.41–2.67)	2.12 (1.39–3.24)
**Perinatal asphyxia**				
** No**	842 (90.7)	187 (98.4)	Reference	
** Yes**	86 (9.3)	3 (1.6)	0.16 (0.05–0.50)	
**Exposure to hemolytic agents/chemicals**				p = 0.012
** No**	917 (98.8)	171 (90.0)	Reference	Reference
** Yes**	11 (1.2)	19 (10.0)	9.26 (4.33–19.81)	3.44 (1.32–8.99)

Model performance: c-stastic: 0.902 (95% CI: 0.879–0.925)

ABE: acute bilirubin encephalopathy; OR: odds ratio; CI: confidence interval; SD: standard deviation

Factors associated with ET are shown in Table 3. Of all infants who received ET 88 (25%) had peak TSB ≤20 mg/dL. All factors, including ABE but excluding weight on admission and sepsis were significantly associated with ET in the univariate analysis. However, only infant’s age on admission, peak TSB, ABO incompatibility and ABE were predictive of ET. Risk of ET was significantly correlated with increasing magnitude of peak TSB. Infants with ABE had significantly higher odds of repeat ET (OR:11.98, 95% CI: 6.44–22.29). These four factors were strongly predictive of ET (c-statistic: 0.925, 95% CI: 0.909–0.940). Male sex was associated with, but not independently predictive of ABE and ET.

**Table 3:** Factors associated with severe hyperbilirubinemia leading to exchange transfusion.

Factors	No ET	ET		
	n = 766 (%)	n = 352 (%)	Unadjusted OR (95% CI)	Adjusted OR (95% CI)
**Gender**				
** Male**	473 (61.7)	244 (69.3)	1.40 (1.07–1.83)	
** Female**	293 (38.3)	108 (30.7)	Reference	
**Age on admission (days)**				p<0.0001
** 1–2**	139 (18.1)	53 (15.1)	1.13 (0.71–1.79)	4.82 (2.37–9.78)
** 3–7**	494 (64.5)	254 (72.2)	1.52 (1.05–2.20)	1.96 (1.17–3.29)
** 8–21**	133 (17.4)	45 (12.7)	Reference	Reference
** Median (Interquartile range)**	4.0 (3.0–7.0)	5.0 (3.0–6.0)		
**Weight on admission (kg)**				
** 2.2 kg or less**	59 (7.7)	38 (10.8)	1.45 (0.94–2.23)	
** Above 2.2kg**	707 (92.3)	314(89.2)	Reference	
** Median (Interquartile range)**	2.9 (2.5–3.3)	2.8 (2.5–3.2)		
**Peak total bilirubin (mg/dL)**				p<0.0001
** <16**	511 (66.7)	16 (4.5)	Reference	Reference
** 16–20**	175 (22.8)	72 (20.5)	13.14 (7.44–23.20)	13.67 (7.41–25.23)
** 21–30**	60 (7.8)	163 (46.3)	86.76 (48.63–154.80)	97.91 (50.57–189.59)
** >30**	20 (2.7)	101 (28.7)	161.28 (80.80–321.93)	149.64 (66.34–333.02)
** Median (Interquartile range)**	13.6 (10.6–16.6)	24.8(20.4–31.5)		
**ABO incompatibility**				p<0.0001
** No**	716 (93.5)	234 (66.5)	Reference	Reference
** Yes**	50 (6.5)	118 (33.5)	7.22 (5.03–10.37)	6.43 (3.82–10.83)
**Rhesus incompatibility**				
** No**	744 (97.1)	333 (94.6)	Reference	
** Yes**	22 (2.9)	19 (5.4)	1.93 (1.03–3.61)	
**Sepsis**				
** No**	532 (69.5)	228 (64.8)	Reference	
** Yes**	234 (30.5)	124 (35.2)	1.24 (0.95–1.62)	
**Perinatal asphyxia**				
** No**	691 (90.2)	338 (96.0)	Reference	
** Yes**	75 (9.8)	14 (4.9)	0.38 (0.21–0.69)	
**Exposure to hemolytic agents/chemicals**				
** No**	753 (98.3)	335 (95.2)	Reference	
** Yes**	13 (1.7)	17 (4.8)	2.94 (1.41–6.12)	
**Acute bilirubin encephalopathy**				p<0.0001
** No**	720 (94.0)	208 (59.1)	Reference	Reference
** Yes**	46 (6.0)	144 (40.9)	10.84 (7.52–15.63)	2.01 (1.25–3.22)

Model performance: c-stastic: 0.925 (95% CI: 0.909–0.940)

ET: exchange transfusion; OR: odds ratio; CI: confidence interval; SD: standard deviation

Factors associated with neonatal mortality are summarized in Table 4. All factors except infant’s gender, age and weight on admission, ABO incompatibility and sepsis were significantly associated with mortality in the univariate analysis. After adjustment for covariates, rhesus incompatibility, asphyxia, exposure to hemolytic substances and ABE were predictive of mortality. Infants who received ET had 69% probability of survival. The discriminatory power of the final model was also strong (c-statistic: 0.922, 95% CI: 0.893–0.957). High bilirubin levels (OR:1.11, 95% CI:1.07–1.14), exposure to hemolytic substances (OR:7.73 95% CI:3.16–18.91) and rhesus incompatibility (OR:6.13 95% CI:2.44–15.43) were predictive of mortality among infants with concomitant ABE (data not shown). Infants with at least one risk factor for neurotoxicity were significantly at risk of ABE (OR:1.97, 95% CI: 1.42–2.72) and hyperbilirubinemia requiring ET (OR:2.35, 95% CI: 1.81–3.05) but not neonatal mortality (OR:1.31, 95% CI: 0.78–2.20) (data not shown).

**Table 4:** Factors associated with mortality among infants admitted for significant neonatal hyperbilirubinemia.

Factors	Alive	Dead		
	n = 1057 (%)	n = 61 (%)	Unadjusted OR (95% CI)	Adjusted OR (95% CI)
**Gender**				
** Male**	673 (63.7)	44 (72.1)	1.48 (0.83–2.62)	
** Female**	384 (36.3)	17 (27.9)	Reference	
**Age on admission (days)**				
** 1–2**	182 (17.2)	10 (16.4)	Reference	
** 3–7**	702 (66.4)	46 (75.4)	1.19 (0.59–2.41)	
** Above 7**	173 (16.4)	5 (8.2)	0.53 (0.18–1.57)	
** Median (Interquartile range)**	5.0 (3.0–7.0)	5.0 (3.0–5.0)		
**Weight on admission (kg)**				
** 2.2 kg or less**	92 (8.7)	5 (8.2)	0.94 (0.37–2.40)	
** Above 2.2kg**	965 (91.3)	56 (91.8)	Reference	
**Median (Interquartile range)**	2.9 (2.5–3.3)	2.9 (2.7–3.0)		
**Peak total bilirubin (mg/dL)**				
** <16**	519 (49.1)	8 (13.1)	Reference	
** 16–20**	241 (22.8)	6 (9.8)	1.62 (0.55–4.71)	
** 21–30**	200 (18.9)	23 (37.7)	7.46 (3.28–16.95)	
** >30**	97 (9.2)	24 (39.3)	16.05 (7.01–36.78)	
** Median (Interquartile range)**	15.7 (11.9–21.5)	28.6 (20.6–31.7)		
**ABO incompatibility**				
** No**	903 (85.4)	47 (77.0)	Reference	
** Yes**	154 (14.6)	14 (23.0)	1.75 (0.94–3.25)	
**Rhesus incompatibility**				p = 0.001
** No**	1026 (97.1)	51 (83.6)	Reference	Reference
** Yes**	31 (2.9)	10 (16.4)	6.49 (3.02–13.96)	5.78 (2.01–16.65)
**Sepsis**				
** No**	713 (67.5)	47 (77.0)	Reference	
** Yes**	344 (32.5)	14 (23.0)	0.62 (0.34–1.14)	
**Perinatal asphyxia**				p<0.0001
** No**	977 (92.4)	52 (85.2)	Reference	Reference
** Yes**	80 (7.6)	9 (14.8)	2.11 (1.01–4.44)	17.0 (5.53–52.26)
**Exposure to hemolytic agents/chemicals**				p<0.0001
** No**	1038 (98.2)	50 (82.0)	Reference	Reference
** Yes**	19 (1.8)	11 (18.0)	12.02 (5.43–26.61)	4.61 (1.72–12.39)
**Acute bilirubin encephalopathy**				p<0.0001
** No**	916 (86.7)	12 (19.7)	Reference	Reference
** Yes**	141 (13.3)	49 (80.3)	26.53 (13.77–51.11)	41.22 (13.54–125.48)
**Had exchange transfusion**				p = 0.002
** No**	737 (69.7)	29 (47.5)	Reference	Reference
** Yes**	320 (30.3)	32 (52.5)	2.54 (1.51–4.27)	0.31 (0.15–0.64)

Model performance: c-stastic: 0.922 (95% CI: 0.893–0.951)

OR: odds ratio; CI: confidence interval

Although infants with severe hyperbilirubinemia were routinely placed under CPT on admission whether or not ET was indicated, no data was available on the duration of treatment and irradiance monitoring of the devices during the study period. Information on the duration of ET, the associated adverse events (if any) and post-exchange TSB levels were not available from the medical records. Public energy supply was frequently unstable necessitating recourse to alternative power sources for all electrical devices including CPT.

## Discussion

This study has shown that hyperbilirubinemia is a significant contributor to neonatal morbidity and mortality in this population. This is consistent with the findings from a recent systematic review of studies published from 1960 to 2014 which suggested that neonatal hyperbilirubinemia may account for up to 64% of neonatal admissions and 13.1% of neonatal mortality [23]. The drop in admissions in 2014 was due to a statewide strike by doctors. While neonatal hyperbilirubinemia remains a significant cause of hospitalization in the first week of life in several countries [4,5,27,28], bilirubin-induced mortality is rare or extremely low in developed countries like USA and Europe [29].

Rhesus incompatibility, exposure to hemolytic substances, perinatal asphyxia, ABE and lack of timely ET emerged as the independent drivers of mortality in this population of infants with hyperbilirubinemia. These findings are consistent with existing literature [10,11,24,29]. Evidence from developed nations has shown that the burden of rhesus disease can be effectively controlled through identification of all Rh-negative women before or during pregnancy and ensuring that they receive Rh immunoprophylaxis postpartum and ideally at the 28th wk of pregnancy within a well-coordinated framework of obstetric and neonatal care [29]. It is noteworthy that Rh incompatibility was not an independent predictor of ABE or severe hyperbilirubinemia requiring ET in this population.

Exposure to icterogenic agents or oxidant stressors such as insecticides, menthol-based baby care products, naphthalene-camphor balls, sulfonamides or sulfa-containing drugs and herbal concoctions have been demonstrated in several studies to trigger severe hyperbilirubinemia and ABE in infants with marked G6PD deficiency [24,30]. The contribution of polymorphism of the uridine-diphosphate-glucuronosyl transferase 1A1 gene (UGT1A1) to the severity of hyperbilirubinemia in these infants has also been reported [31]. Although we were unable to establish the incidence of G6PD deficiency in our study population, the high prevalence of G6PD deficiency in our country (estimated national frequency: 16.9%, 95% interquartile range: 14.1–20.2) makes it plausible to implicate G6PD deficiency in the elevated risk of ABE and mortality among infants exposed to hemolytic substances [23].

The observation regarding perinatal asphyxia is well-established in the literature and remains a global health priority for developing countries in general. The Helping Babies Breathe initiative spearheaded by the American Academy of Pediatrics and the American Heart Foundation is currently being systematically disseminated nationwide [32]. The current and future impact of this intervention in our hospital deserves a separate evaluation.

Perhaps the most overarching finding from this report is the potential impact of controlling the high rates of ABE and ET on neonatal mortality. Our study (Table 3) suggests that curtailing avoidable ET requires timely detection and management of infants with rising TSB levels, those with neurotoxic risk factors like ABO incompatibility and those with or at risk of ABE. Reducing the incidence of ABE itself, would require attention to infection control and management as well as exposure to hemolytic substances. Late presentation also has to be addressed because of poor maternal recognition of severe hyperbilirubinemia and cultural predisposition to traditional therapies before seeking medical attention [11]. The role of prenatal maternal education in these efforts cannot be over-emphasized [33]. The risk of ABE is well-established for preterm or low birth weight infants [34].

Although ET was a protective factor against mortality, the high rates are of concern in this resource-constrained setting. Timely provision of effective phototherapy reduces the need for ET. Evidence from comparable studies in LMICs suggests that excessive rates of ET are attributable to several factors that include late presentation to health facilities, inadequate diagnostic and monitoring facilities and sub-optimal phototherapy [11,24,35–37]. At our center, the provision of effective CPT was frequently hampered by unstable power source, frequent device breakdowns, sub-optimal irradiance and the overwhelming demand on our limited CPT devices by the number of infants who present with severe hyperbilirubinemia with or without referrals.

Efficient laboratory support remains an essential component of care [38]. However, complete diagnostic work-up was frequently hampered by lack of modern laboratory facilities. For example, routine quantitative or qualitative tests for G6PD would have been helpful in profiling jaundiced infants for the risk of hemolysis. So also is the provision of side laboratory in the neonatal unit with facilities for real-time TSB measurement and monitoring. For example, not all infants who were transfused developed ABE, underscoring the crucial value of timely TSB monitoring. In fact, it is not unusual to find some infants with very high TSB levels without evidence of any hemolytic disease or ABE [39]. Essential clinical and laboratory support must therefore, be ensured to optimize the benefits of the proposed intervention. The requisite tasks and facilities have been detailed in a recent review [40].

A major merit of this paper is the insights provided on critical indices for evaluating the impact of FSPT on the care of jaundiced infants in this hospital beyond the safety and efficacy evaluation of the device itself. However, some limitations of this retrospective study are worth noting. Firstly, like most clinical chart reviews, the available information was limited to what was considered necessary or attainable at the point of care. As a result some relevant data such as the G6PD status and maintenance records including irradiance levels of the phototherapy devices were not available. Secondly, the diagnostic validity of some of the variables could not be independently evaluated, although no spurious results emerged from our analysis. Thirdly, it was difficult to evaluate the degree of compliance with the treatment protocol over the study period. Fourthly, we could not establish baseline data for adverse events associated with ET or the pattern of post-exchange TSB levels. Notwithstanding, the key findings are indicative of issues that need to be appropriately addressed in a prospective study to facilitate objective and reliable evaluation of the impact of FSPT in this and any comparable resource-constrained population [41].

## Conclusions

This study has shown that infants with hyperbilirubinemia account for a significant proportion of neonatal admissions yearly and are associated with substantial rates of ABE, avoidable ET and premature death yearly. Several of the contributory factors are amenable to intervention including the provision of effective phototherapy. FSPT must therefore seek to curtail the prevailing adverse outcomes frequently associated with infants presenting in this hospital to make the intervention worthwhile.

## Abbreviations

ABE: Acute bilirubin encephalophathy
CI: Confidence interval
CPT: Conventional blue-light phototherapy
ET: Exchange transfusion
FSPT: Filtered Sunlight Phototherapy
G6PD: Glucose 6-phospho-dehydrogenase
IQR: Interquartile range
LMICs: Low- and middle-income countries
MSCH: Massey Street Children’s Hospital
OR: Odds ratio
TSB: Total serum/plasma bilirubin

## References

[1] National Institute for Health and Clinical Excellence. Neonatal jaundice. (Clinical guideline 98.), 2010. www.nice.org.uk/CG98. Accessed 25 October 2015.

[2] Watchko JF, Tiribelli C. Bilirubin-induced neurologic damage—mechanisms and management approaches. *N Engl J Med* 2013;369:2021–2030. 10.1056/NEJMra1308124 24256380

[3] Maisels MJ. Managing the jaundiced newborn: a persistent challenge. *CMAJ* 2015;187:335–343. 10.1503/cmaj.122117 25384650 PMC4361106

[4] The Young Infants Clinical Signs Study Group. Clinical signs that predict severe illness in children under age 2 months: a multicentre study. *Lancet* 2008;371:135–142. 10.1016/S0140-6736(08)60106-3 18191685

[5] Burke BL, Robbins JM, Bird TM, Hobbs CA, Nesmith C, Tilford JM. Trends in hospitalizations for neonatal jaundice and kernicterus in the United States, 1988–2005. *Pediatrics* 2009;123:524–532. 10.1542/peds.2007-2915 19171618

[6] Steiner LA, Bizzarro MJ, Ehrenkranz RA, Gallagher PG. A decline in the frequency of neonatal exchange transfusions and its effect on exchange-related morbidity and mortality. *Pediatrics* 2007;120:27–32. 17606558 10.1542/peds.2006-2910

[7] Chitty HE, Ziegler N, Savoia H, Doyle LW, Fox LM. Neonatal exchange transfusions in the 21st century: a single hospital study. *J Paediatr Child Health* 2013;49:825–832. 10.1111/jpc.12290 23834341

[8] Abu-Ekteish F, Daoud A, Rimawi H, Kakish K, Abu-Heija A. Neonatal exchange transfusion: a Jordanian experience. *Ann Trop Paediatr* 2000;20:57–60. 10824215 10.1080/02724930092084

[9] Salas AA, Mazzi E. Exchange transfusion in infants with extreme hyperbilirubinemia: an experience from a developing country. *Acta Paediatr* 2008;97:754–758. 10.1111/j.1651-2227.2008.00743.x 18422806

[10] Owa JA, Ogunlesi TA. Why we are still doing so many exchange blood transfusion for neonatal jaundice in Nigeria. *World J Pediatr* 2009;5:51–55. 10.1007/s12519-009-0009-2 19172333

[11] Olusanya BO, Ogunlesi TA, Slusher TM. Why is kernicterus still a major cause of death and disability in low-income and middle-income countries? *Arch Dis Child* 2014;99:1117–1121. 10.1136/archdischild-2013-305506 25123403

[12] Basnet S, Adhikari N, Koirala J. Challenges in setting up pediatric and neonatal intensive care units in a resource-limited country. *Pediatrics* 2011;128:e986–992. 10.1542/peds.2010-3657 21930539

[13] Narang A, Kiran PS, Kumar P. Cost of neonatal intensive care in a tertiary care center. *Indian Pediatr* 2005;42:989–997. 16269829

[14] Opondo C, Ntoburi S, Wagai J, Wafula J, Wasunna A, Were F, et al Are hospitals prepared to support newborn survival?–An evaluation of eight first-referral level hospitals in Kenya. *Trop Med Int Health* 2009;14:1165–1172. 10.1111/j.1365-3156.2009.02358.x 19695001 PMC2751740

[15] Bhutani VK, Cline BK, Donaldson KM, Vreman HJ. The need to implement effective phototherapy in resource-constrained settings. *Semin Perinatol* 2011;35:192–197. 10.1053/j.semperi.2011.02.015 21641494

[16] Pejaver RK, Vishwanath J. An audit of phototherapy units. *Indian J Pediatr* 2000; 67:883–884. 11262986 10.1007/BF02723951

[17] Cline BK, Vreman HJ, Faber K, Lou H, Donaldson KM, Amuabunosi E, et al Phototherapy device effectiveness in Nigeria: irradiance assessment and potential for improvement. *J Trop Pediatr* 2013; 59:321–325. 10.1093/tropej/fmt027 23666953

[18] Slusher TM, Vreman HJ, Olusanya BO, Wong RJ, Brearley AM, Vaucher YE, et al Novel treatment of neonatal jaundice: safety and efficacy of filtered sunlight in African neonates. *Pediatrics* 2014;133:e1568–1574. 24864170 10.1542/peds.2013-3500 PMC4531268

[19] Slusher TM, Olusanya BO, Vreman HJ, Brearly AM, Vaucher YE, Lund TC, et al A randomized trial of filtered sunlight phototherapy in African neonates. *New Engl J Med* 2015; 373:1115–1124. 10.1056/NEJMoa1501074 26376136

[20] Olusanya B, Imam Z, Mabogunje C, Emokpae A, Slusher T. Maternal satisfaction with a novel filtered-sunlight phototherapy for newborn jaundice in Southwest Nigeria. *BMC Pediatr* 2014;14:180 10.1186/1471-2431-14-180 25012576 PMC4099408

[21] Cheesbrough M. District Laboratory Practice in Tropical Countries. 2nd ed. Cambridge, UK: Cambridge University Press; 2006:362–378.

[22] World Health Organization. Pocket book of hospital care for children: Guidelines for the management of common childhood illnesses. First & Second editions. Geneva: WHO 2005, 2013. 24006557

[23] Olusanya BO, Emokpae AA, Zamora TG, Slusher TM. Addressing the burden of severe neonatal hyperbilirubinaemia in low and middle-income countries with significant G6PD-deficiency. *Acta Paediatr* 2014;103:1102–1109. 10.1111/apa.12735 24990658

[24] Olusanya BO, Osibanjo FB, Mabogunje CA, Slusher TM, Olowe SA. The burden and management of neonatal jaundice in Nigeria: a scoping review of the literature. *Niger J Clin Pract* 2016;19:1–17. 10.4103/1119-3077.173703 26755212

[25] American Academy of Pediatrics (AAP). Management of hyperbilirubinaemia in the newborn infant 35 or more weeks of gestation. *Pediatrics* 2004;114:297–316. 15231951 10.1542/peds.114.1.297

[26] Hanley JA, McNeil BJ. The meaning and use of the area under a Receiver Operating Characteristic (ROC) curve. *Radiology* 1982;143:29–36. 7063747 10.1148/radiology.143.1.7063747

[27] Habib HS. Impact of discharge timings of healthy newborns on the rates and etiology of neonatal hospital readmissions. *J Coll Physicians Surg Pak* 2013;23:715–719. 10.2013/JCPSP.715719 24112257

[28] Calado CS, Pereira AG, Santos VN, Castro MJ, Maio JF. What brings newborns to the emergency department?: a 1-year study. *Pediatr Emerg Care* 2009;25:244–248. 19382329 10.1097/pec.0b013e31819e361d

[29] Bhutani VK, Zipursky A, Blencowe H, Khanna R, Sgro M, Ebbesen F, et al Neonatal hyperbilirubinemia and Rhesus disease of the newborn: incidence and impairment estimates for 2010 at regional and global levels. *Pediatr Res* 2013;74 Suppl 1:86–100. 10.1038/pr.2013.208 24366465 PMC3873706

[30] Slusher TM, Vreman HJ, McLaren DW, Lewison LJ, Brown AK, Stevenson DK. Glucose-6-phosphate dehydrogenase deficiency and carboxyhemoglobin concentrations associated with bilirubin-related morbidity and death in Nigerian infants. *J Pediatr* 1995;126:102–108. 7815196 10.1016/s0022-3476(95)70510-4

[31] Kaplan M, Slusher T, Renbaum P, Essiet DF, Pam S, Levy-Lahad E, et al (TA) n UDP-glucuronosyltransferase 1A1 promoter polymorphism in Nigerian neonates. *Pediatr Res* 2008;63:109–111. 18043502 10.1203/PDR.0b013e31815b8e7e

[32] Disu EA, Ferguson IC, Njokanma OF, Anga LA, Solarin AU, Olutekunbi AO, et al National neonatal resuscitation training program in Nigeria (2008–2012): a preliminary report. *Niger J Clin Pract* 2015;18:102–109. 10.4103/1119-3077.146989 25511353

[33] Zhang L, Hu P, Wang J, Zhang M, Zhang QL, Hu B. Prenatal Training Improves New Mothers' Understanding of Jaundice. *Med Sci Monit* 2015;21:1668–73. 26056164 10.12659/MSM.893520 PMC4471851

[34] Ogunlesi TA, Dedeke IO, Adekanmbi AF, Fetuga MB, Ogunfowora OB. The incidence and outcome of bilirubin encephalopathy in Nigeria: a bi-centre study. *Niger J Med* 2007;16:354–9. 18080595

[35] Hameed NN, Na' Ma AM, Vilms R, Bhutani VK. Severe neonatal hyperbilirubinaemia and adverse short-term consequences in Baghdad, Iraq. *Neonatology* 2011;100:57–63. 10.1159/000321990 21212697

[36] Rasul CH, Hasan MA, Yasmin F. Outcome of neonatal hyperbilirubinemia in a tertiary care hospital in Bangladesh. *Malays J Med Sci* 2010;17:40–44. PMC3216155 22135536

[37] Iskander I, Gamaleldin R, Kabbani M. Root causes for late presentation of severe neonatal hyperbilirubinaemia in Egypt. *East Mediterr Health J* 2012;18:882–887. 23057379 10.26719/2012.18.8.882

[38] Mabogunje CA, Olaifa SM, Olusanya BO. Facility-based constraints to exchange transfusions for neonatal hyperbilirubinemia in resource-limited settings. *World J Clinical Pediatrics* 2016 (in press). 10.5409/wjcp.v5.i2.182 PMC4857231 27170928

[39] Gamaleldin R, Iskander I, Seoud I, Aboraya H, Aravkin A, Sampson PD, et al Risk factors for neurotoxicity in newborns with severe neonatal hyperbilirubinemia. *Pediatrics* 2011;128:e925–e931. 10.1542/peds.2011-0206 21911352 PMC3182847

[40] Olusanya BO, Ogunlesi TA, Kumar P, Boo NY, Iskander IF, de Almeida MF, et al Management of late-preterm and term infants with hyperbilirubinaemia in resource-constrained settings. *BMC Pediatrics* 2015;15:39 10.1186/s12887-015-0358-z 25884679 PMC4409776

[41] Hess DR. Retrospective studies and chart reviews. *Respir Care* 2004;49:1171–1174. 15447798

